# Targeted next generation sequencing identified novel mutations in *RPGRIP1* associated with both retinitis pigmentosa and Leber’s congenital amaurosis in unrelated Chinese patients

**DOI:** 10.18632/oncotarget.17052

**Published:** 2017-04-12

**Authors:** Hui Huang, Ying Wang, Huishuang Chen, Yanhua Chen, Jing Wu, Pei-Wen Chiang, Ning Fan, Yan Su, Jianlian Deng, Dongna Chen, Yang Li, Xinxin Zhang, Mengxin Zhang, Shengran Liang, Santasree Banerjee, Ming Qi, Xuyang Liu

**Affiliations:** ^1^ BGI-Shenzhen, Shenzhen, China; ^2^ Shenzhen Key Laboratory of Ophthalmology, Shenzhen Eye Hospital, Jinan University, Shenzhen, China; ^3^ Key Laboratory of Optoelectronic Devices and Systems of Ministry of Education and Guangdong Province, College of Optoelectronic Engineering, Shenzhen University, Shenzhen, China; ^4^ School of Bioscience and Bioengineering, South China University of Technology, Guangzhou, China; ^5^ Casey Eye Institute Molecular Diagnostic Laboratory, Portland, Oregon, USA; ^6^ BGI Education Center, University of Chinese Academy of Sciences, Shenzhen, China; ^7^ Department of Applied Biology with Chemical Technology, The Hong Kong Polytechnic University, Hung Hom, Kowloon, Hong Kong; ^8^ School of Basic Medical Sciences, Zhejiang University, Hangzhou, China; ^9^ Functional Genomics Center, Department of Pathology & Laboratory Medicine, University of Rochester Medical Center, West Henrietta, New York, USA; ^10^ School of Ophthalmology & Optometry, Shenzhen University, Shenzhen, China

**Keywords:** targeted next generation sequencing, gene panel, novel mutation, retinitis pigmentosa, Leber's congenital amaurosis

## Abstract

As the most common inherited retinal degenerations, retinitis pigmentosa (RP) is clinically and genetically heterogeneous. Some of the *RP* genes are also associated with other retinal diseases, such as LCA (Leber's congenital amaurosis) and CORD (cone-rod dystrophy). Here, in our molecular diagnosis of 99 Chinese RP patients using targeted gene capture sequencing, three probands were found to carry mutations of *RPGRIP1*, which was known to be associated with pathogenesis of LCA and CORD. By further clinical analysis, two probands were confirmed to be RP patients and one was confirmed to be LCA patient. These novel mutations were co-segregated with the disease phenotype in their families. Our result not only expands the mutational spectrum of the *RPGRIP1* gene but also gives supports to clinical diagnosis and molecular treatment of RP patients.

## INTRODUCTION

Retinitis pigmentosa (RP) is the most common inherited retinal dystrophy, affecting approximately 1 in 5,000 individuals worldwide [1, 2]. RP is primarily associated with the rod photoreceptors, while the cone cells are compromised as the disease progresses [3]. RP individuals exhibit degeneration of photoreceptors or the retinal pigment epithelium (RPE) of the retina in the initial stages of the disease, followed by a progressive reduction in the visual field and visual loss [3]. Other ocular findings include atrophic changes in the photoreceptors and RPE followed by the appearing of melanin-containing structures in the retinal vascular layer [3]. The fundus changes include a pale optic nerve, attenuated retinal vessels, and bone spicule-like pigmentation in the mid-peripheral retina [3].

RP is phenotypically and genetically heterogeneous with almost 60 different genes identified till now, and could be divided into the early onset RP which exhibits symptoms around 2 years old and the late onset RP which may not develop symptoms until mid-life [2]. The clinical distinction between the subtypes of RP, however, is not always clear and there are largely overlapped in phenotype between RP, LCA (Leber's congenital amaurosis), COD or CORD (cone or cone-rod dystrophy) and other retinal diseases [3]. Also, there are other non-RP diseases caused by mutations in the same genes involved with RP. With the development of sequencing technology, more disease-causing genes have been identified, 60 for RP, 20 for LCA and 29 for CORD respectively. Among them, 9 genes (*CRB1, CRX, LRAT, PRPH2, RDH12, RPE65, SPATA7, TULP1, IMPDH1*) were previously reported to be associated with both RP and LCA (RetNet). However, large number of RP patients is waiting for uncovering their specific genetic underlying causes. In our recent molecular screening of 99 RP patients, two RP and one LCA patients were found to carry mutated *RPGRIP1*, which was previously reported to be implicated in LCA and CORD [4, 5].

## RESULTS

### Clinical findings

**Figure 1:**
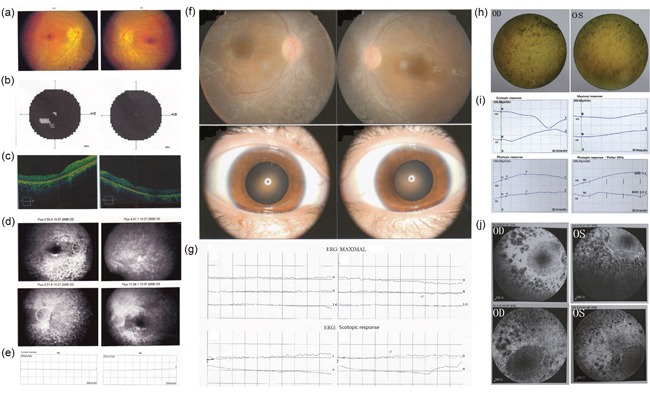
Clinical characteristics of 3 patients Patient P065 **(a)** Fundus photographs showed disc pallor with wax yellow. Obviously attenuated narrow arterioles and tortuous veins lay on the orange background of the retina. **(b)** vision field examination (Topcon SBP-300) show tubular vision with litter center visual island. **(c)** OCT showed significant thinner of outer layer of retina, especially in the left eye. **(d)** Fudus fluorescence angiography (FFA) showed a significant delay in the arm-retina circulation time (ART) from 10˜15sec to 17sec of the left eye. There were uniform scattered dotted transmitted fluorescences on the both side in the late phase. Absent peripheral background fluorescence indicted choriocapillaris were atrophy. Patchy blocked fluorescence existed in the corresponding pigmentary area in the inferior periphery retina of the right eye. The normal structure of macular arch was absent. **(e)** Scotopic ERG (rod response) and dark adapted bright flash ERG showed a-, b-waves extinguished in both eyes. The patient exhibited extinguished in the ERG response. Patient P024 **(f)** Fundoscopy revealed narrowing of retinal vessels. Scattered patchy dislocatedpigmentary changes could be seen in the periphery retina of both eyes. **(g)** ERG revealed extinguished waveforms. Patient P030 **(h)** Dilated fudus examination revealed arterioles and tortuous veins narrowed. Patient's optic disk was sanguineness. Obviously bone corpuscle types pigmentary were seen in the periphery retina. **(i)** Scotopic ERG (rod response) after 30min dark adaptation showed the extinguishment of waveform in both eyes. B-wave of photopic ERG (cone response) after 10min light adaptation showed implicit time delay to 49ms which was amplified to severe decline in both eyes. Patient exhibited the absence of nomal ERG response to a 30Hz flickering light. **(j)** FFA showed Retinal arterioles were narrowed. The edges of optic disks were vague. The illumination of periphery scattered transmitted fluorescences was increasing gradually. Patchy blocked fluorescence existed in the periphery area. Vessels obstruction induced massive periphery non-perfusion area.

Patient P065 is a 30 years old male with early-onset RP. He exhibited early-onset bilateral blurred vision in childhood (visual acuity: 20/25), nystagmus and night blindness at about 23 years old based on his recall. Then he gradually presented with decreasing vision and loss of peripheral visual fields. At 30 years of age, his best corrected visual acuity (BCVA) was 20/133 with -2.25 diopter in the right eye and 20/200 with -2.00 diopter in the left. Fundus examination revealed waxy disc, obviously attenuated retina vascular. No significant pigmentary changes of salt and pepper or bone corpuscle type were noted in the left eye. A few patchy pigmentary could be seen inferior in the periphery retina (Figure 1a). Center 30° visual field examination (Topcon SBP-300) showed tubular vision with little center visual island left (Figure 1b). Time-dominant optical coherence tomography (TD-OCT) showed significant thinner outer retina, especially in the left eye (Figure 1c). Fundus fluorescence angiography (FFA) showed a significant delay in the arm-retina circulation time (ART) from 10˜15sec to 17sec of the left eye, which exhibited considerable attenuation of the retinal arterioles. Absent peripheral background fluorescence indicated that choriocapillaris were atrophied. Patchy blocked fluorescence existed in the corresponding pigmentary area in the inferior periphery retina of the right eye. The normal structure of macular arch was absent (Figure 1d). Full-field electroretinography (fERGs) disclosed no detectable rod responses to single flashes of blue light and cone responses to the 30 Hz flicker that were reduced 99% and delayed (Figure 1e & Supplementary Figure 1). All the status of severe retinal structure abnormity and function loss support the clinical diagnosis of RP.

Patient P024, as diagnosed with RP is a 22 years old female with progressive vision weakness for 20+ years. She is the only patient in a non-consanguineous family. Nystagmus was noticed in her first 8 months, and then gradual loss of visual acuity and night blindness appeared. In the last 6 years, her visual field showed losses of peripheral vision, and eventually became tunnel vision in the age of 21. Her BCVA was 12/400 with both eyes. The dilated fundus examination showed attenuated retina vascular with absent pigmentary changes (Figure 1f). The ERG performed extinguished waveform, which indicated that the rod cell and cone cell were severely damaged (Figure 1g).

Patient P030 is female, 36 years of age, who was diagnosed as LCA. She was born with severe visual impairment and nystagmus without any other symptoms. Her BCVA was hand motion in the age of 36. Dilated fundus examination revealed narrowed arterioles and tortuous veins. Obviously bone corpuscle types pigmentary were seen in the periphery retina (Figure 1h). Scotopic ERG (rod response) after 30min dark adaptation showed the extinguishment of the a-wave and b-wave in both eyes. Only three waves of oscillatory potential ERG were recorded, their amplitudes were severe declined. Cone system was affected with slightly delayed b-wave implicit time. He exhibited the absence of normal ERG response to a 30Hz flickering light (Figure 1i). Fundus fluorescence angiography (FFA) showed the edges of optic disks were vague. The illumination of periphery scattered transmitted fluorescences was gradually increasing. Patchy blocked fluorescence existed in the periphery area. Vessels obstruction induced massive periphery non-perfusion area (Figure 1j).

### Molecular diagnosis and validation

We performed next generation sequencing on these 3 patients using the panel described in Methods section. 466, 362, and 351 fold of depth, 94.26%, 94.31%, and 94.04% of coverage were obtained from the 3 patient samples (Supplementary Table 1), respectively, indicating that sufficient depth and coverage were obtained for variants calling. We identified 8, 15, 8 rare variants (rare: frequency < 0.01 in 1000genome, dbSNP, HGMD and our 200 control local database) that would lead to protein coding change (including potential splice site in 10bp of exon/intron borders) in the 283 eye disease related genes (Supplementary Table 2).

**Figure 2:**
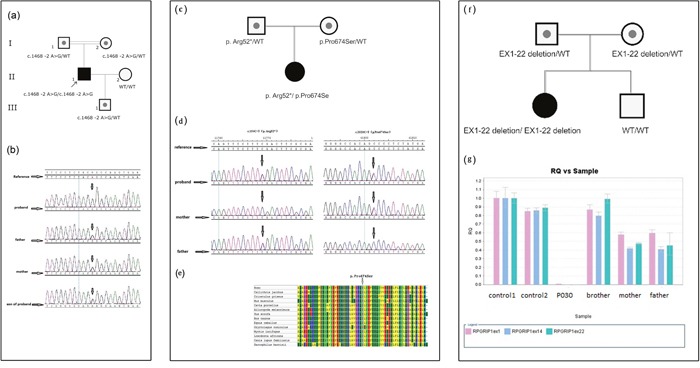
Molecular genetic test **(a)** The pedigree of Patient P065; **(b)** Sanger sequence chromatograms for the pedigree of Patient P065; **(c)** The pedigree of patient P024; **(d)** Sanger sequence chromatograms for the pedigree of Patient P024; **(e)** Evaluation of the novel *RPGRIP1* missense mutation in Patient P024. Multiple alignments using MEGA6 software (Tamura K et al. 2013) and amino acid conservation of the novel missense sequence variants were performed. The alignment results showed that proline at codon 2020 were fully conserved through all species. **(f)** The pedigree of patient P030; **(g)** Quantity PCR for the large deletion in pedigree of Patient P030.

We found these 3 patients carrying mutations not in RP related genes but in gene *RPGRIP1* which was previously reported to cause Leber's congenital amaurosis [4] and Cone-rod dystrophy [5]. Among them, one patient carried compound heterozygous mutations and 2 patients carried homozygous mutations (Table 1). Sanger sequencing and quantitative PCR was undertaken to validate all these 4 *RPGRIP1* mutations in the proband and their family members (Figure 2).

**Table 1:** *RPGRIP1* mutations identified in three families

Patient ID	Disease	Type	Exon	Mutation	Protein change
65	ARRP	Homozygous	Intron11	c.1468-2A>G	Exon12 del
24	ARRP	Compound heterozygous	Exon 2 / Exon 14	c.154C>T/c.2020C>T	p.Arg52*/ p.Pro674Ser
30	LCA	Homozygous	Exon1-22	ex1-22del	

RP: Retinitis Pigmentosa; LCA: Leber congenital amaurosis.

Patient P065 was detected to carry one novel homozygous splicing site mutation c.1468-2A>G in gene *RPGRIP1*, his parents were consanguineous with normal phenotype. This homozygous mutation was not recorded in the HapMap and 1K genome and was co-segregated with the disease phenotype in the family; the normal family members carried only one heterozygous mutant allele (Figure 2a & Figure 2b).

This *RPGRIP1* mutation disrupts the splice acceptor site of exon 12. With this type of splicing defect, it would be desirable to determine the consequences at the mRNA level by reverse transcription-PCR (RT-PCR) of *RPGRIP1* cDNA. Partial genomic DNA constructs consisting of exons 12 and partial intron 11 and intron 12 of *RPGRIP1*, with or without the splice site mutation, were expressed in COS-7 cells. RT-PCR and direct sequencing of *RPGRIP1* cDNA from cells transfected with wild-type construct showed normal splicing of exon 12. In contrast, direct sequencing of mutant RT-PCR products revealed complete loss of exon 12 resulting removal of 48 amino acids in the RPGRIP1 polypeptide due to abolition of the acceptor splice site (data not shown). Prediction of the abolition effect of this splice site was made using a splice predictor program, Human Splicing Finder (http://www.umd.be/HSF). This prediction gave a potential breakage of splice site (ttccctctacggCC), compared with a probability of 92.13 for the wild type (ttccctctacagCC) splice site. All the results suggest that aberrant splicing caused by this mutation is quite efficient as it completely abolishes normal splicing in the mutant allele.

Patient P024 carried one nonsense mutation c.154C>T (p. Arg52*) and one novel missense mutation c.2020C>T (p. Pro674Ser) in gene *RPGRIP1*. Mutation p. Arg52* was inherited from the father and mutation c.2020C>T (p. Pro674Ser) came from the mother (Figure 2c & Figure 2d). The nonsense mutation p. Arg52* was previously reported in a retinitis punctata albescens (RPA) patient [6]. It creates premature stop codons and is likely to be a null allele. The mutation p. Pro674Ser is novel and not recorded in the HapMap and 1K genome database or our local database, it changed a proline (non-polar, uncharged amino acid) into a serine (polar) in the protein. In addition, it was predicted to be “probably damaging” with a score of 1.000 using PolyPhen-2 (http://genetics.bwh.harvard.edu/pph2/). The proline at position 674 is present as a highly conserved amino acid among mammals (Figure 2e). It is highly likely that these mutations are the primary cause of the retinal disease in this patient.

Patient P030 was found carrying no SNV/InDel mutation, so we tried to search a CNV (copy number variantion) from exonic depth and coverage in retinal genes, and a large homozygous deletion of exon 1-22 in *RPGRIP1* was found (Supplementary Figure 2), which was inherited from her healthy parents separately (Figure 2f). The subsequent quantity PCR confirmed this mutation (Figure 2g). This large deletion may lead to absence of RPGRIP1 protein which interacts with RPGR in the connecting of cilia of rods and cones [7]. This homozygous mutation was not identified in the HapMap and 1000 genome and was co-segregated with the disease phenotype. Her normal brother didn't carry the mutation (Figure 2g).

## DISCUSSION

In this study, we found 2 RP patients and 1 LCA patient carrying mutations in *RPGRIP1* using our panel NGS methods. One RP patient carried a novel homozygous mutation of c.1468-2A>G in intron 11 of *RPGRIP1*, resulting in the formation of alternative transcripts and leading to the complete loss of exon 12 (data not shown). Defects in either RPGRIP1 or its RPGR interacting protein probably alter a functional complex in the connecting cilia of rods and cones. Another RP patient carried compound heterozygote for c.154C>T (p. Arg52*) and c.2020C>T (p. Pro674Ser). The LCA patient carried a large homozygous deletion that extends from exon 1 to intron 22 in *RPGRIP1*. All these mutations were confirmed to be co-segregated with disease phenotype in respective families by Sanger sequencing or quantitative PCR.

This study provides evidence supporting that *RPGRIP1* is a new RP-causing gene by finding mutations in 2 unrelated cases. We also found that the LCA patient carrying mutation in *RPGRIP1* presented with similar phenotype. In clinical signs and symptoms, all these 3 patients presented with early-onset night blindness and low visual acuity. According to the clinical examination and genetic test of these 3 patients, it suggested a correlation between disease severity and the nature of the mutations. Patient P030 carrying a large exon1-22 deletion mutation in RPGRIP1 showed the most severe phenotype of three patients, she was blind with light perception at birth and her current BCVA was hand motion, and she was diagnosed with LCA. Patient P024 and P065, who were diagnosed with RP, showed slighter symptoms than Patient P030. Patient P024 carrying a nonsense (p. Arg52*) and a missense mutation (p. Pro674Ser) showed a worse BCVA comparing to Patient P065 who carried a homozygous splice site mutation (c.1468-2A>G). It is possible that the large proportion of coding region deletion and nonsense mutation in the initiating terminal coding region leads to loss of the whole protein, while the missense mutation and the splice site mutation (although it resulted in the complete loss of exon 12) retains most of protein residue.

The *RPGRIP1* gene contains 24 exons and encompasses three main domains that are RPGR interacting domain (RID), coiled-coil (C2) domains and a domain of unknown function. The RPGRIP1 protein is localized in the connecting cilia of human cone and rod photoreceptors that connect inner and outer segments, where it binds RPGR to the cilium. Mice lacking *RPGRIP* elaborate grossly oversized outer segment disks resembling a cytochalasin D-induced defect and have a more severe disease than mice lacking RPGR. Mice lacking both proteins are phenotypically indistinguishable from mice lacking RPGRIP alone [8]. RPGRIP1 has an interaction not only with RPGR, but also with *NPHP4* and *SDCCAG8*, Rpgrip1^nmf247^ mice without *RPGRIP1* expression lack NPHP4 and RPGR in photoreceptor cilia, whereas the SDCCAG8 and acetylated-a-tubulin ciliary localizations are strongly decreased, even though the NPHP4 and SDCCAG8 expression levels are normal and those of acetylated-a-tubulin and c-tubulin are upregulated [9]. Mutations in *NPHP4* and *SDCCAG8* would cause Senior-Loken syndrome and Bardet-Biedl syndrome, these two disorders are characterized by LCA/RP and other systemic features including kidney conditions [10–11]. These studies suggest that RPGRIP1 is important in visual pathway and has an essential function in eye development, and when mutated, it may cause RP disease.

**Figure 3:**
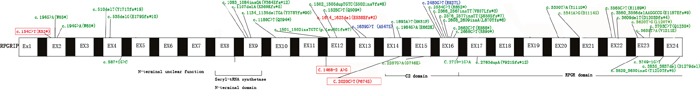
Reported variants in *RPGRIP1* identified in individuals with LCA, CORD and RP

Interestingly, RP is both clinically and genetically heterogeneous. The phenotype of such patients is overlap between retinal diseases, as RP and LCA is more overlap in the initial stage, while RP and CORD is in the end stage [3]. There are nine genes (*CRB1, CRX, LRAT, PRPH2, RDH12, RPE65, SPATA7, TULP1, IMPDH1*) overlap between RP and LCA, eight genes (*CRX, PROM1, PRPH2, SEMA4A, ABCA4, C8orf37, CERKL, RPGR*) between RP and COD/CORD (RetNet: https://sph.uth.edu/retnet/sum-dis.htm). *CRX* and *PRPH2* are the only two genes associated with RP, LCA and CORD. Carlo Rivolta et al. analyzed 18 mutations and Huang L et al. also reviewed 49 mutations in *CRX*, but they all could not uncover any correlation between phenotype and genotype [12–13]. In this study, we also summarized the mutations in *RPGRIP1* which were reported to be related to LCA or CORD as many as we can (Figure 3). We can see that approximately 90% of the reported mutations causing LCA in *RPGRIP1* were frameshift or nonsense mutations that will lead to create a prematurely truncate protein, which are expected to abolish the function of RPGR interacting domain, even the RPGRIP1 function. *RPGRIP1* was found to cause LCA in 2001 [4], then in 2002, Cremers FP et al. deduced that mutations allow residual RPGRIP1 activity may result in less severe phenotypes, such as RP or CORD, than LCA [14]. Subsequently in 2003, it was proved that homozygous missense mutations in *RPGRIP1* would cause CORD [5]. In 2005, J C Booij et al. found two RP patients carrying respective frameshift and missense mutations in *RPGRIP1*, but it's a pity that they identified only one heterozygous mutation in every patient [15]. Yet, in this study, we found two RP patients caused by homozygous splice site mutation and compound heterozygous (missense and nonsense) mutations in *RPGRIP1*. Taken together, it showed strong evidence for the conclusion that *RPGRIP1* is a causative gene for RP.

In the past, people always found new disease-causing genes by linkage study and subsequent whole exome sequencing (WES). In this study, we found a new RP disease-causing gene by panel sequencing. Although all the captured genes were known hereditary eye disease-causing genes, many different retinal disease genes are in the same pathway (KEGG), it provides the potential possibility that one gene in this pathway causes several retinal diseases. So we can find a new gene for some retinal diseases using a panel that capture genes in the pathway.

In conclusion, we reported that the mutations in *RPGRIP1* causes early-onset RP based on the data from panel NGS, co-segregation, OCT, histopathological and electrophysiological analysis. Thus our findings suggest that *RPGRIP1* gene can also be classified into the RP causative genes, that expands the clinical spectrum of *RPGRIP1* related disease. Since the two RP patients in this study were found in a cohort of 99 unselective Chinese patients, we can expect that *RPGRIP1* would cause about 2% RP patients in Chinese population. In addition, *RPGRIP1* has been reported with effective treatment in animal models and showing molecular treatment potential in a LCA patient [16, 17]. Hope our research could improve the diagnosis and treatment of RP and LCA.

## MATERIALS AND METHODS

### Patients

This study was performed in agreement with the declaration of Helsinki. All the patients and their legal representatives have given permission for the mutation detection. Informed consents were also obtained from patients. This study was approved by the institutional review board on bioethics and biosafety of BGI, and the IRB approval number is BGI-IRB 14002.

### DNA preparation

Venous blood samples were obtained from the probands and their family members. Genomic DNA was extracted from 200μl peripheral venous blood by standard procedures using QIAmp DNA Blood Mini kit (QIAGEN, Hilden, Germany) according to recommended instructions. DNA integrity was evaluated by 2% agarose gel electrophoresis. All DNA samples were stored at -20°C after the analysis with NanoDrop 2000 (Thermo Fisher Scientific, Waltham, USA).

### Panel sequencing library preparation and sequencing

283 genes, which include 164 known retinal disease-causing genes, were collected by systematic database (GeneReviews(®), OMIM, and RetNet), literature searches, and expert reviews (Supplementary File 1). Customized oligonucleotide probes were designed to capture exons and adjacent 30bp sequences of above genes using NimbleGen (Roche) online oligonucleotide probe design system. Target sequencing libraries were prepared as below: 1μg genomic DNA was sonicated to 200˜300 bp fragments, followed with end-repair, A-tailing, Illumina adaptors ligation, 4 cycles of pre-capture PCR amplification and sample indexing. Then the indexed PCR product of 20-30 samples were pooled, targeted capture was performed by hybridizing with capture probes, and followed by 15 cycles of PCR amplification. DNA sequencing was done on Illumina HiSeq2500 sequencer to generate paired-end reads including 90 bps at each end and 8 bps of the index tag. And bases were called by the Illumina build-in Pipeline.

### Bioinformatics analysis and data filtering

Reads filtering was surveillance by indexed primers. The reference genome was downloaded from the NCBI, version GRCh37 (hg19). Sequence was aligned to the reference using Burrows Wheeler Aligner (BWA) Multi-Vision software package [18]. SNVs were called using SOAPsnp [19], while small InDels (<20bp) were called using the Samtools (Tools for alignments in the SAM format) Version: 0.1.18, http://samtools.sourceforge.net/. The threshold for filtering SNPs included the followings: 1) the consensus quality score had to be >= 20 (The quality score is a Phred score, generated by the program SOAPsnp, quality score 20 represents 99% accuracy of a base call); 2) the number of uniquely mapped reads supporting a SNP had to be >= 4; 3) the estimated copy number is no more than 2; 4) the distance between two SNPs should be larger than 5.

### Sanger sequencing

Mutations identified in Target sequencing were validated by Sanger sequencing. Primers flanking the candidate loci were designed based on reference genomic sequences of Human Genome from gene bank in NCBI (GRCh37, hg19) and synthesized by Invitrogen (Shanghai, China). PCR amplification was carried out in ABI 9700 Thermal Cycler. Subsequently, all PCR products were sequenced on ABI PRISM 3730 automated sequencer (Applied Biosystems).

### Minigene construction and exon-trapping

To determine the function of the mutation at transcriptional level, *in vitro* exon trapping studies were performed. Using DNA from proband as a template, harboring a homozygous mutant allele, a genomic fragment, containing 315bp of intron 11, exon 12 and 312bp of intron 12 with both 5’ and 3’ intronic flanking sequences, was generated and cloned into pSPL3 (Invitrogen, Carlsbad, CA) exon trapping vector through double digestion by *Bam*HI and *Xho*I. WT and mutant plasmids were transiently transfected into COS-7 (African green monkey kidney fibroblast-like) cell line using lipo2000 (Invitrogen, Carlsbad, CA). COS-7 cells were cultured in Dulbecco's modified Eagle's medium supplemented with 10% fetal bovine serum, 1% penicillin-streptomycin, and 1% glutamine in a humidified, 5% CO2 incubator at 37°C. At 48h post transfection, total RNA was extracted with Trizol (TaKaRa, Dalian, China). cDNA was prepared using 5 μg total RNA in a total volume of 20 μl with superscript II RNAse H-reverse transcriptase and oligo-dT priming (TaKaRa, Dalian, China). Amplification products obtained by PCR with vector primers SD6 (5’-TCTGAGTCACCTGGACAACC-3’) and SA2 (5’-ATCTCAGTGGTATTTGTGAGC-3’), with recommended PCR reaction condition, were separated on a 2% TBE agarose gel. Amplification products were characterized by direct sequencing.

## SUPPLEMENTARY MATERIALS TABLES




