# Supplementary material for: Targeted next generation sequencing identified novel mutations in RPGRIP1 associated with both retinitis pigmentosa and Leber’s congenital amaurosis in unrelated Chinese patients
Source: Oncotarget. 2017 Apr 12;8(21):35176–83. doi: 10.18632/oncotarget.17052 (PMC5471044; doi:10.18632/oncotarget.17052)
Supplement: Supplementary file 1 [file oncotarget-08-35176-s001.pdf]

Targeted next generation sequencing identified novel mutations in *RPGRIP1* associated with both retinitis pigmentosa and Leber's congenital amaurosis in unrelated Chinese patients

SUPPLEMENTARY FIGURES, TABLES AND MATERIAL

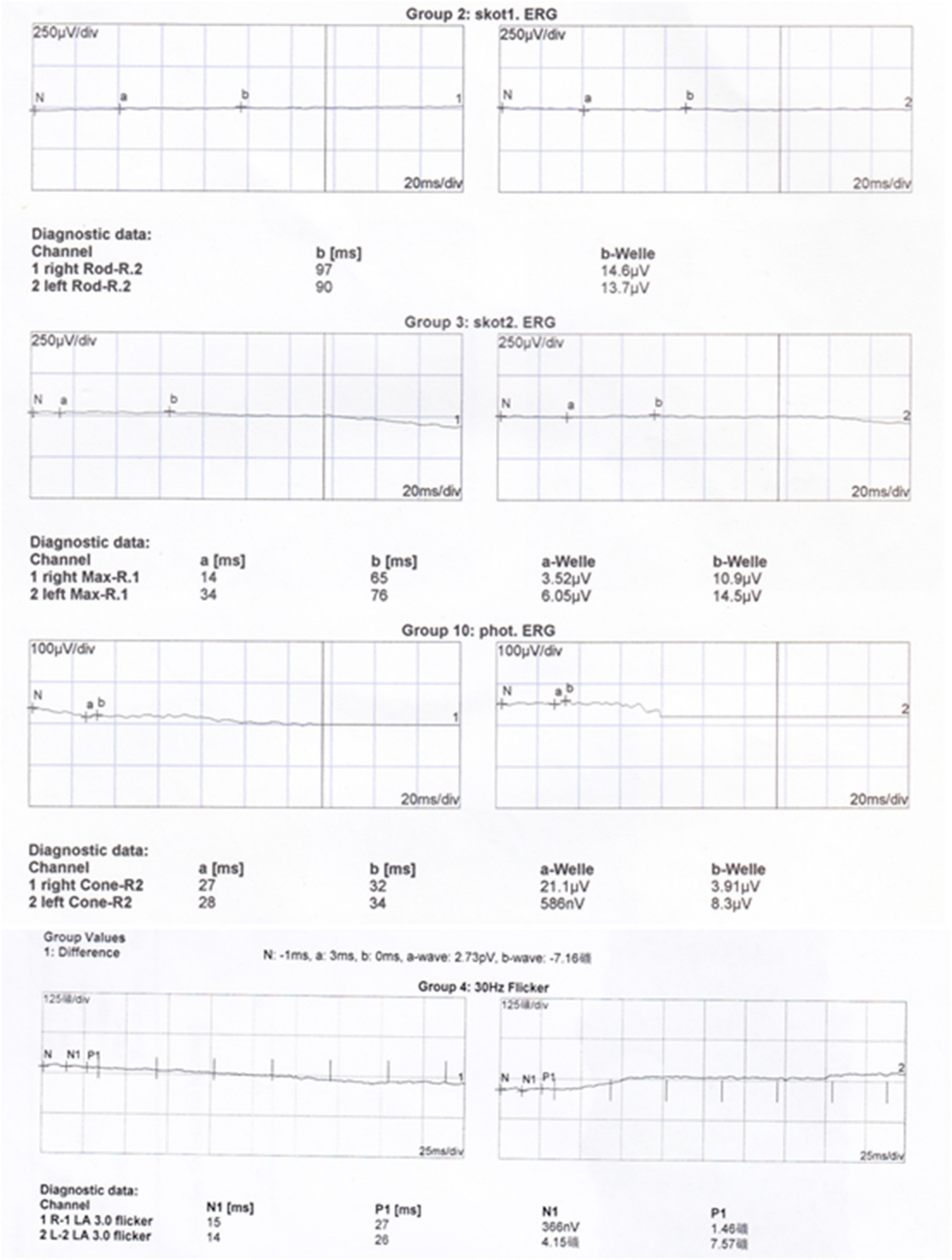

Supplementary Figure 1: ERG of Patient P065.

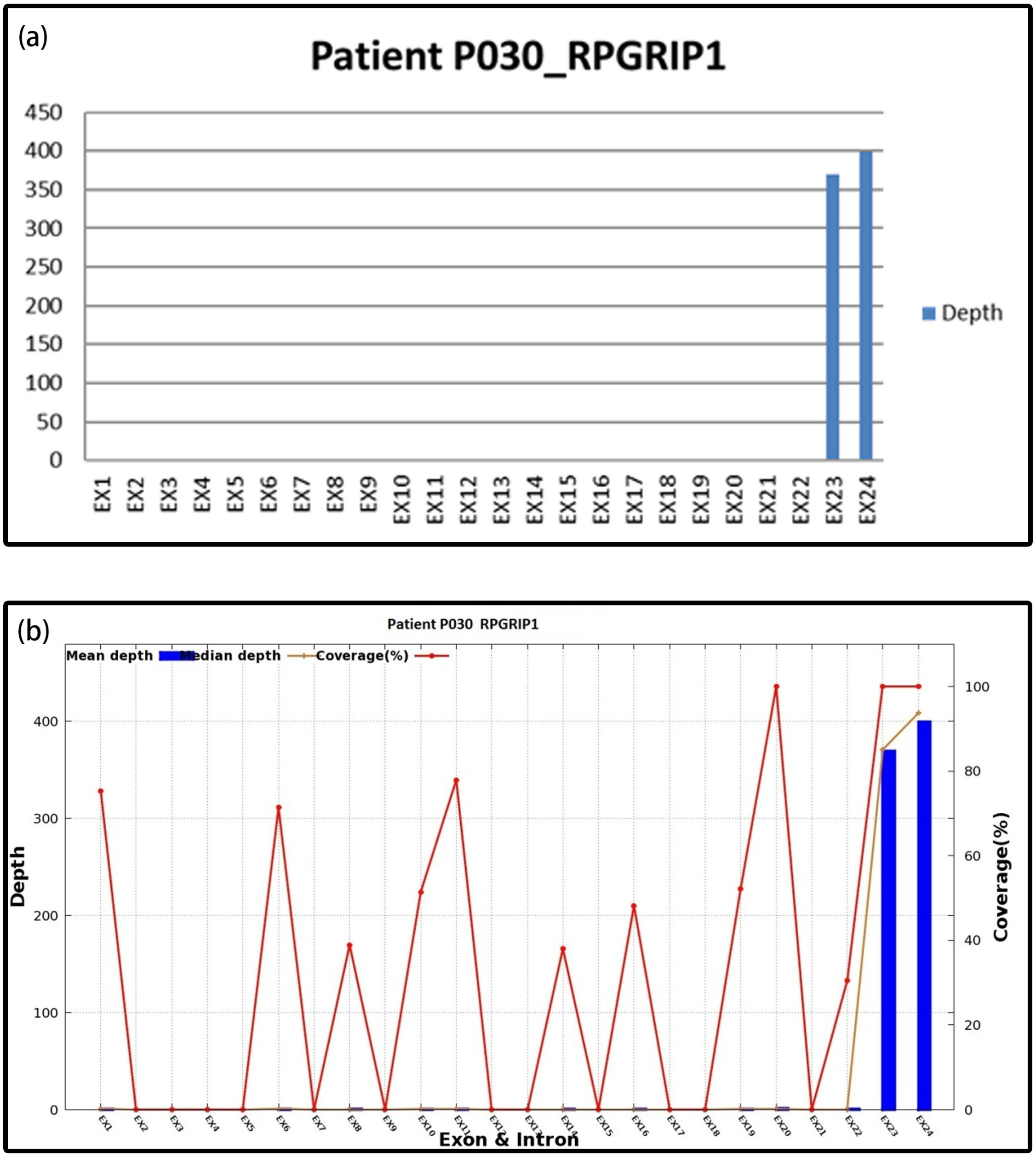

Supplementary Figure 2: The sequencing depth of exon1-22 in *RPGRIP1* in patient 30 is zero fold, while exon23-24 is nearly 400 fold.

Supplementary Table 1: Panel NGS statistics for the three patients

Index	Patient P 065	Patient P024	Patient P 030
Raw reads (maped to hg19)	14,347,756	11,325,674	10,961,330
Raw data yield (Mb)	1,291.30	1,019.31	986.52
Reads mapped to target region	77.29%	78.68%	79.55%
Data mapped to target region	57.40%	56.59%	56.57%
Coverage of target region	94.26%	94.31%	94.04%
Average sequencing depth of target region	465.76	362.47	350.7
Target region covered with at least 4X	93.79%	93.76%	93.45%
Target region covered with at least 10X	93.32%	93.32%	93.00%
Target region covered with at least 20X	92.79%	92.85%	92.49%

Supplementary Table 2: Rare variants that would lead to protein coding change

Chromosome	Gene	Het/Hom	Mutation name	Residue Change	Frequency in 1k genome	Frequency in local database
			**Patient P065**			
chr1	NPHP4	Het	c.3045 -5 C>T	_	0.0037	0
chr2	ALMS1	Het	c.3724G>C	p.Glu1242Gln	0	0
chr2	PAX3	Het	c.86 -6 C>G	_	0	0
chr9	TYRP1	Het	c.1571A>C	p.Glu524Ala	0.0009	0
chr14	RPGRIP1	Hom	c.1468 -2 A>G	_	0	0
chr16	ZNF469	Het	c.4423C>G	p.Leu1475Val	0.0055	0
chr17	FSCN2	Het	c.1441G>T	p.Gly481Cys	0	0
chr14	PABPN1	Het	c.223_223delC	_	0	0
			**Patient P024**			
chr1	HMCN1	Het	c.6917G>A	p.Arg2306Gln	0.0018	0.0051
chr1	HMCN1	Het	c.11938G>A	p.Val3980Met	0.0073	0
chr1	USH2A	Het	c.6524G>A	p.Arg2175His	0.0027	0
chr2	MERTK	Het	c.1441C>T	p.Pro481Ser	0	0
chr8	RP1L1	Het	c.3992T>G	p.Leu1331Arg	0	0
chr8	RP1L1	Het	c.3991C>G	p.Leu1331Val	0	0.0051
chr8	RP1L1	Het	c.3989G>A	p.Gly1330Glu	0	0.0051
chr8	RP1L1	Het	c.3983A>C	p.Glu1328Ala	0	0
chr10	CDH23	Het	c.68 -3 C>T	_	0.0055	0
chr10	CDH23	Het	c.1282G>A	p.Asp428Asn	0.0009	0
chr11	MYO7A	Het	c.6092G>A	p.Arg2031Gln	0	0
chr12	CEP290	Het	c.4697C>T	p.Ala1566Val	0	0
chr14	RPGRIP1	Het	c.154C>T	p.Arg52*	0.0009	0
chr14	RPGRIP1	Het	c.2020C>T	p.Pro674Ser	0	0
chr21	COL18A1	Het	c.2326_2334delCCCCCTGGG	_	0	0.0051
			**Patient P030**			
chr1	HMCN1	Het	c.11927C>T	p.Ala3976Val	0	0
chr2	SNRNP200	Het	c.5024C>T	p.Ala1675Val	0	0
chr2	MERTK	Het	c.1282A>G	p.Ser428Gly	0	0.0051
chr5	GPR98	Het	c.3884G>A	p.Gly1295Asp	0	0
chr11	MYO7A	Het	c.5582G>A	p.Arg1861Gln	0	0
chr12	MLL2	Het	c.11398G>A	p.Gly3800Ser	0	0
chr16	ZNF469	Het	c.9896C>T	p.Pro3299Leu	0.0037	0
chr20	IDH3B	Het	c.908T>C	p.Phe303Ser	0	0

Supplementary Materials 1:

See Supplementary File 1
